# Effect of Sub-Zero Treatments and Tempering on Corrosion Behaviour of Vanadis 6 Tool Steel

**DOI:** 10.3390/ma14133759

**Published:** 2021-07-05

**Authors:** Peter Jurči, Aneta Bartkowska, Mária Hudáková, Mária Dománková, Mária Čaplovičová, Dariusz Bartkowski

**Affiliations:** 1 Institute of Materials Science, Faculty of Materials Science and Technology in Trnava, Slovak University of Technology, J. Bottu 25, 917 24 Trnava, Slovakia; maria.hudakova@stuba.sk (M.H.); maria.domankova@stuba.sk (M.D.); 2 Institute of Materials Science and Engineering, Faculty of Materials Engineering and Technical Physics, Poznan University of Technology, ul. Jana Pawła II 24, 61-138 Poznan, Poland; aneta.bartkowska@put.poznan.pl; 3 Laboratories of STU Centre for Nanodiagnostics, Slovak University of Technology in Bratislava, Vazovova 5, 812 43 Bratislava, Slovakia; maria.caplovicova@stuba.sk; 4 Institute of Materials Technology, Faculty of Mechanical Engineering, Poznan University of Technology, ul. Piotrowo 3, 61-138 Poznan, Poland; dariusz.bartkowski@put.poznan.pl

**Keywords:** ledeburitic steel, sub-zero treatment, microstructure, potentiodynamic polarisation, corrosion resistance

## Abstract

Sub-zero treatment of Vanadis 6 steel resulted in a considerable reduction of retained austenite amount, refinement of martensite, enhancement of population density of carbides, and modification of precipitation behaviour. Tempering of sub-zero-treated steel led to a decrease in population density of carbides, to a further reduction of retained austenite, and to precipitation of M_3_C carbides, while M_7_C_3_ carbides precipitated only in the case of conventionally quenched steel. Complementary effects of these microstructural variations resulted in more noble behaviour of sub-zero-treated steel compared to the conventionally room-quenched one, and to clear inhibition of the corrosion rate at the same time.

## 1. Introduction

Chromium-vanadium (Cr-V) ledeburitic steels are widely used as materials for the manufacturing of the powder compaction dies, seamless tube pilgering, press tools, paper cutters, extruders, and metal cutting tools, because of the attractive combination of the hardness, wear resistance, and toughness. Generally, these materials obtain their properties through appropriate heat treatment. The standard heat treatment of Cr-V ledeburitic tool steels is comprised of austenitizing, quenching, and double (or triple) tempering, which results in a hardness of around 60 HRC, high compressive strength, excellent wear resistance, and relatively good toughness when properly treated.

Sub-zero treatment (SZT) was introduced to the industries in the late 1950s. This kind of treatment is defined as a process which is carried out at temperatures from 0 to −269 °C. The main benefits of the SZT have been reported as elevated hardness [1,2,3,4], additional wear resistance [2,3,5,6,7,8,9,10,11], and better dimensional stability [12]. In some cases, the toughness and fracture toughness also manifested certain, but very limited, improvement [6,13].

The above-mentioned ameliorations originate from several sources. The latest investigations arrived at general conclusions, finding that the application of SZT results in the following four main microstructural changes [1,9,11,14,15,16,17,18,19]:

- Sub-zero-treated materials contain significantly reduced retained austenite (γ_R_) amounts, as a result of isothermal and time-dependent martensitic transformation, which takes place during the SZT.
- The martensite of SZT steel is refined compared to that developed by conventional room-temperature quenching.
- Sub-zero treatment leads to acceleration of precipitation of transient carbides during either the re-heating to room temperature or short-term storage at room temperature.
- The last consequence of the SZT is the considerably enhanced number and population density of small globular carbides (SGCs). The formation of the SGCs at cryogenic temperature is time-dependent and follows the reduction of the retained austenite amount well.

The corrosion resistance is not commonly considered as a key property for tool materials. This is due to the fact that the dominant number of technological applications is conducted in corrosion-friendly environments, or, if not, there exist surfacing techniques (such as physical vapour deposition, for instance) through which a sufficiently high corrosion resistance of tools can be ensured. Despite this, it might be desirable to keep at least acceptable corrosion behaviour of tools made of Cr and Cr-V ledeburitic tool steels in some applications, where, for instance, surface techniques fail for several reasons. These applications may comprise industrial branches, such as mining, earth-handling, milling, powder compaction, mineral processing, etc. Here, high processing reliability and sufficient tools’ durability require materials with not only excellent wear performance but also with at least acceptable corrosion resistance.

However, the characterisation of the effect of SZT on the corrosion behaviour of Cr and Cr-V ledeburitic steels is almost completely lacking. There are only a few relevant studies published on this topic, and the obtained results manifest clear inconsistency. Amini et al. [20] reported on the worsened corrosion behaviour of 1.2080 grade (AISI D3) steel due to the SZT in liquid nitrogen for 24–48 h. According to their consideration, the worsening of corrosion resistance can be ascribed to the increased carbide percentage, which decreases the number of solutionised chromium atoms in the martensite, as well as to the increased martensite/carbide interfaces (galvanic cell areas). Hill et al. [21], on the contrary, recorded an improvement of corrosion resistance of sub-zero-treated (−196 °C/15 min) X190CrVMo 20-4 ledeburitic steel when tested in 0.5 molar sulphuric acid solution.

The present study attempts to overcome the limitation of the lack of data on the corrosion behaviour of sub-zero-treated ledeburitic tool steels. It describes the effects of different sub-zero treatment temperatures (−75, −140, −196, and −269 °C) and tempering regimes on the corrosion behaviour of Cr-V ledeburitic steel Vanadis 6. Conventionally treated steel has been used as a reference. In addition, the relationships between the microstructures, heat treatment parameters, and corrosion behaviour are presented and thoroughly discussed.

## 2. Materials and Experimental Methods

### 2.1. Materials and Processing

The tool steel Vanadis 6, with chemical composition as shown in Table 1, was used for the examinations.

**Figure 1:**
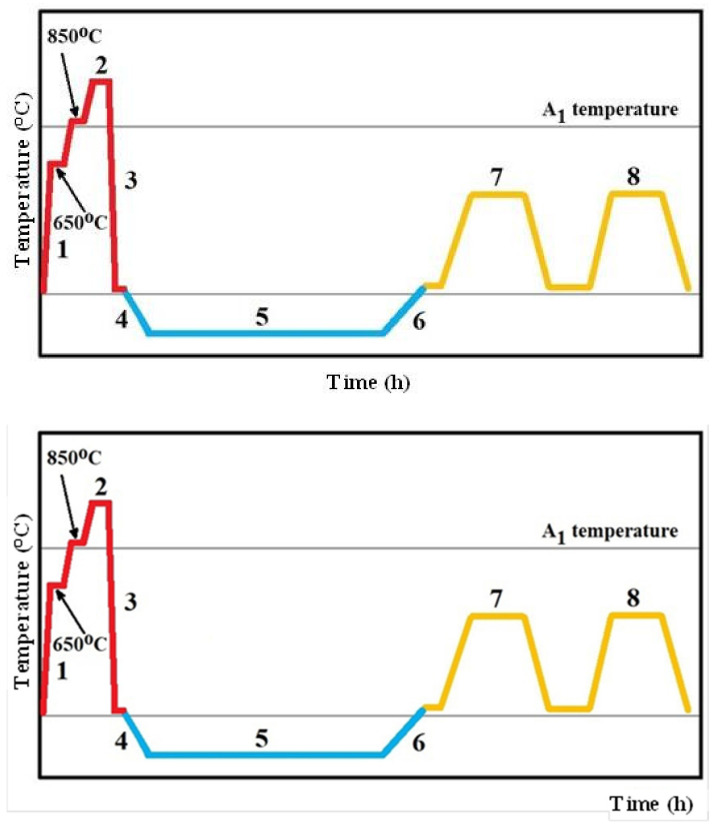
A schematic of the heat treatment schedules used.

Specimens of the studied steel with a size of 20 mm × 20 mm × 7 mm were gradually heated (step (1) in Figure 1) up to the austenitizing temperature of 1050 °C in a vacuum, held there for 30 min (2), and quenched by nitrogen gas to room temperature (3). Then, the specimens were divided to five batches. The first one was immediately subjected to tempering treatment (7, 8). This treatment route is called conventional heat treatment, CHT. Immediately after quenching, the other four sets were moved to the cryogenic system, where they were cooled down at a cooling rate of 1 °C/min to a pre-determined SZT temperature (4). The SZTs were carried out at temperatures of −75, −140, −196, or −269 °C, and for 17 h each (5). After that, the material was re-heated to room temperature, at a heating rate of 1 °C/min (6). Immediately after re-heating, sub-zero-treated specimens were subjected to tempering. Tempering was carried out at temperatures of 170, 330, 450, or 530 °C, 2 times each, for 2 h (7, 8).

### 2.2. Experimental Methods

The specimens for the microstructural examinations were mounted in conductive resin, ground on silicon carbide papers up to 1600 grit, and then progressively polished with 9, 3, and 1 μm diamond paste. The etchant used was 3% ethanol solution of picric acid. The secondary electron (SE) micrographs were acquired with a JEOL JSM 7600 F (Jeol Ltd., Tokyo, Japan) high-resolution field emission scanning electron microscope (SEM), operating at an acceleration voltage of 15 kV. The microstructural examinations were coupled with semi-quantitative analysis of chemical composition of phases by using energy-dispersive spectroscopy (EDS). The basis of the carbide particles’ classification and the determination of their quantitative characteristics were elaborated on earlier, and are demonstrated in recent papers [14,22]. In brief, the eutectic carbides (ECs) are vanadium-based MC-particles, hence, they differ from the secondary M_7_C_3_-carbides (SCs) in terms of their chemistry. Therefore, EDS analysis was used to differentiate between these two carbide types. In order to differentiate between the small globular carbides (SGCs) and other particles, a classification according to their size was used, and the particles smaller than 500 nm were then denoted as SGCs. For the determination of the population densities of ECs, SCs, and SGCs, 25 randomly acquired SEM micrographs, at a magnification of 3000×, were used. Then, the mean values and standard deviations were calculated from the obtained experimental data.

Semi-quantitative chemical composition of phases of sub-micron size (metallic elements only) and the microstructure of both the martensite and the austenite were examined by transmission electron microscopy (TEM). Thin foils for TEM were prepared by sectioning specimens, with a special saw, to obtain pieces with a thickness of 0.1 mm, which were then ground mechanically on silicon carbide paper (1200 grit) to a thickness of approximately 0.02 mm. Final thinning was carried out using an electro-polisher Struers-Tenupol 5. A TEM JEM ARM 200 cF (Jeol Ltd., Tokyo, Japan) equipped with an energy-dispersive spectroscopy (EDS) detector was used for acquisition of micrographs as well as for the determination of phase compositions.

The phases in differently heat-treated specimens were identified from the X-ray diffraction (XRD) profiles by using a Phillips PW 1710 diffractometer. A filtered Co_α1,2_ characteristic radiation, obtained at the voltage of 40 kV and current of 40 mA, has been used for acquisition of diffraction line profiles, within a range of 37–130° of two-theta angles. The analysis was coupled with Rietveld refinement of the X-ray line profiles. The retained austenite amount was determined following the ASTM E975-13 standard [23].

Corrosion resistance studies were carried out by the potentiodynamic polarisation measurements (TAFEL). The TAFEL measurements were completed by using the potentiostat-galvanostat ATLAS 0531 EU (Atlas-Sollich, Rębiechowo, Poland) and IA ATLAS SOLLICH (Atlas-Sollich, Rębiechowo, Poland). A platinum electrode was used as an auxiliary electrode, while a calomel electrode was used as the reference electrode and tested specimens as the working electrodes. The data were recorded by AtlasCorr (Atlas-Sollich, version 3.19) and AtlasLab (Atlas-Sollich, version 2.24) computer software. After corrosion tests, the tested specimens were examined by using SEM coupled with EDS.

Before testing, all the specimens were ground using metallographic emery papers with a grit size up to 2000, and finally, were polished using 3 μm diamond slurry. Just prior to measurements, the specimens were degreased in ethanol and warm air-dried. The 3.5 (in mass %) NaCl water solution was prepared using distilled water and high-purity reagent grade sodium chloride. Then, the solution was subjected to Ar gas bubbling for 30 min in order to its deaerate. For the corrosion tests, the temperature of the solution was kept constant at 22 °C.

The potentiodynamic polarisation measurements were carried out within the range of potentials of −1.5 to 1.5 V, and at a scan rate of 1 mV/s.

The corrosion rate for each specimen was estimated according to the ASTM G 102-89 standard [24]. The calculation was based on the validity of the Faraday’s Law, and it followed Equation (1):

(1) $$C_{R}=K_{1}\cdot \frac{i_{cor}}{\rho }\cdot E_{W}$$

where *C_R_* is the corrosion rate (mm/year), *I_cor_* is the current density (µA/cm^2^), *ρ* is the specific density of the alloy (g/cm^3^), *K*_1_ = 3.27·10^−3^, (mm × g/µA × cm × year), and *E_W_* is the equivalent weight of the experimental steel.

Equivalent weight, *E_W_*, represents the mass of metal, in grams, that will be oxidised by the passage of one Faraday of electric charge. The value of *E_W_* of the experimental material was calculated upon its known chemical composition, atomic weight of the major elements, *W*, the most common valence of a particular element *n*, and as a reciprocal value of the sum of the electron equivalents of all the major elements, *Q*, following Equations (2) and (3):

(2) $$E_{W}=\frac{1}{\sum \frac{n_{i}\cdot f_{i}}{W_{i}}}$$

(3) $$Q=\sum \frac{n_{i}\cdot f_{i}}{W_{i}}$$

where *f_i_* is the mass percentage of the *i*th element in the alloy, *W_i_* is the atomic weight of the *i*th element in the alloy, and *n_i_* is the most common valence of the *i*th element of the alloy. All the input data for the calculations are collected in Table 2. Additionally, it should be noted that an assumption of corrosion uniformity was adopted in the present study, in order to simplify the considerations.

In order to analyse the differences in nobility between the carbides and matrix, the Kelvin probe force microscopy (KPFM) has been adopted. This technique enables to distinguish between local potentials of different phases, at the sub-micron level. Hence, it is suitable for analysing fine-grained PM ledeburitic steels. The analyses were performed at The University of Manchester, Department of Materials Corrosion, by using a Multimode 8 instrument (Bruker), in the amplitude modulated mode. The imaging parameters were the following: 50 × 50 μm scans, potential maps obtained from a 50 nm lift height, using a 0.3 Hz scan rate, 512 points per line, 256 lines, and height images obtained using 100 nm peak force amplitude.

## 3. Results

### 3.1. Microstructure

**Figure 2:**
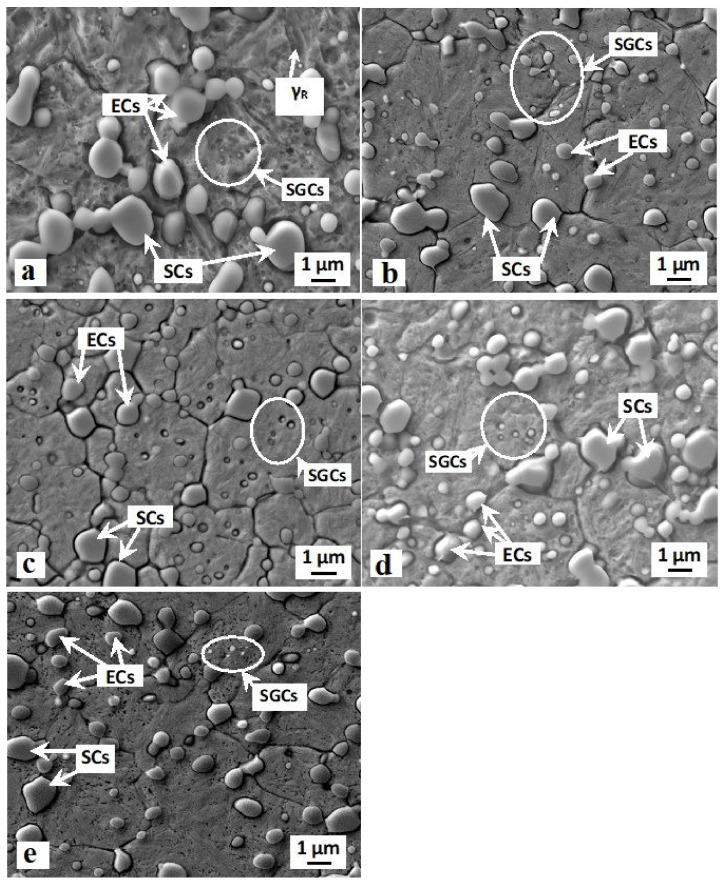
Scanning electron microscopy (SEM) micrographs showing the microstructure of Vanadis 6 ledeburitic steel after conventional room temperature quenching (**a**) and after SZT at −75 °C (**b**), −140 °C (**c**), −196 °C (**d**), and −269 °C (**e**).

SEM images (Figure 2) show the microstructures of the Vanadis 6 steel obtained by the conventional quenching and by different sub-zero treatments. The steel after conventional room temperature quenching is composed of martensitic matrix with the presence of a relatively large retained austenite amount, and of three carbide types (Figure 2a). The carbides are, according to the classification reported in [14]: eutectic carbides (ECs), secondary carbides (SCs), and small globular carbides (SGCs). The character of the matrix microstructure does not change significantly with the application of sub-zero treatment, at the magnification used for the SEM observations (Figure 2b–e). The formations of retained austenite become almost invisible after SZT, due to the significant reduction of *γ*_R_ amount due to this kind of treatment. Alternatively, sub-zero-treated steel differs from that after CHT in terms of the number and population density of carbide particles (compare the SEM micrograph in Figure 2a with micrographs in Figure 2b–e). This concerns mainly the SGCs: the population density of these particles is much higher after SZT, while the SZT does not alter the population densities of ECs and SCs, as demonstrated recently [4,13,19].

**Figure 3:**
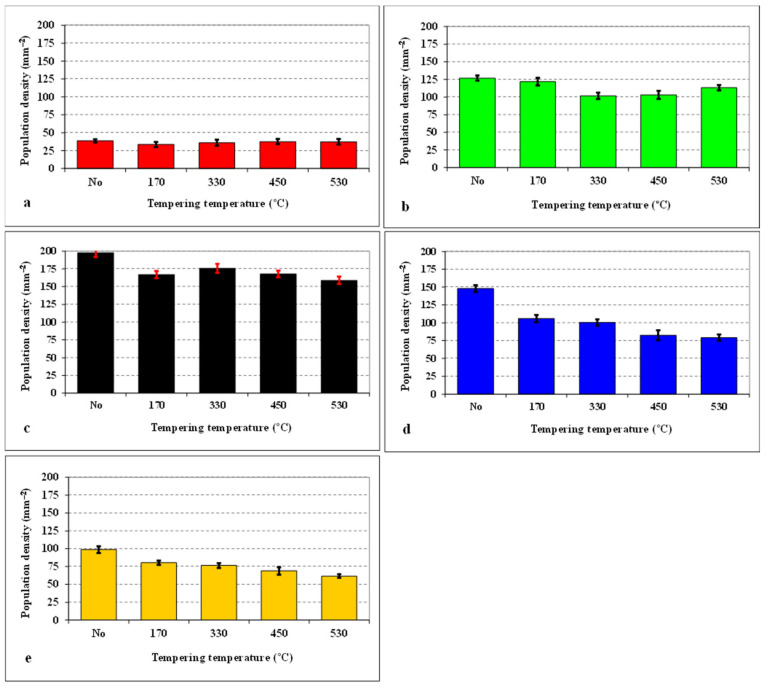
Population density of small globular carbides (SGCs) for differently sub-zero treated (SZT) specimens as a function of tempering temperature: (**a**) conventional heat treatment (CHT), (**b**) SZT −75 °C, (**c**) SZT −140 °C, (**d**) SZT −196 °C, (**e**) SZT −269 °C.

Figure 3 summarizes the population densities of SGCs, which were obtained by different combinations of SZT and tempering. It is shown that the tempering after CHT does not influence the population density of these particles, while the tempering after SZT makes their population density lower than that obtained by SZT without tempering. Despite this fact, the population density of SGCs is much higher than what can be obtained by CHT and tempering. It is also shown that the application of SZT at −140 °C acts in the most effective way in the enhancement of the SGCs population density, and the population densities obtained by other sub-zero treatments are much lower at equal tempering temperatures.

**Figure 4:**
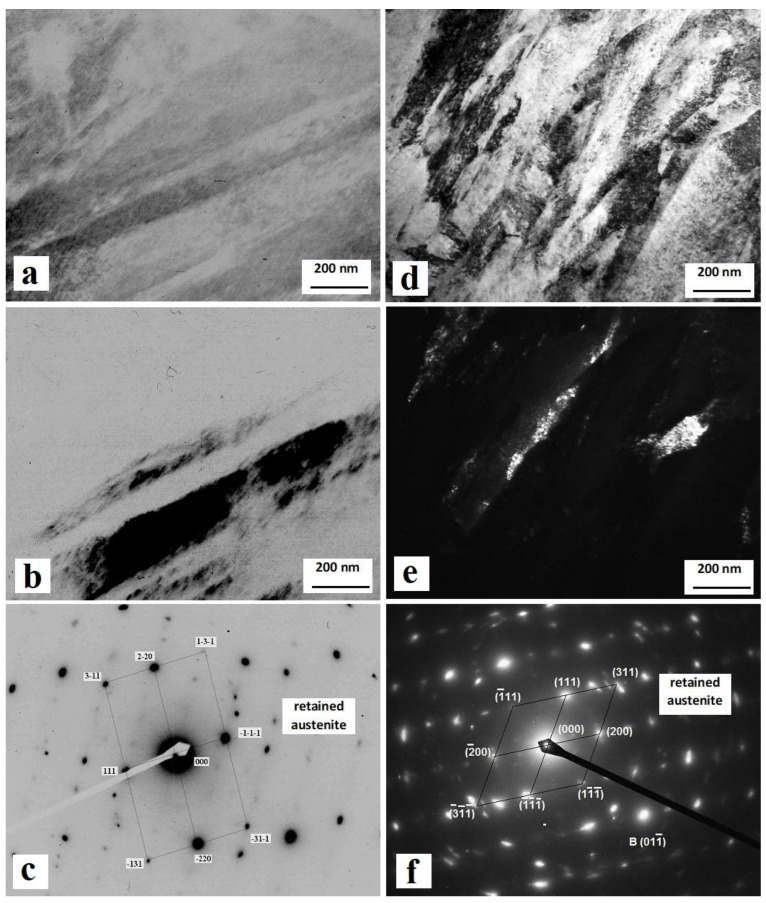
Transmission electron microscopy (TEM) micrographs of the CHT specimen (**a**–**c**) and the specimen that was subjected to SZT at −196 °C (**d**–**f**). (**a**) Bright-field image showing martensitic needle microstructure with retained austenite at needle interfaces, (**b**) corresponding dark-field image showing the retained austenite, (**c**) diffraction patterns of the retained austenite, (**d**) bright-field image showing martensitic microstructure with a small amount of retained austenite at the interfaces of martensitic domains, (**e**) corresponding dark-field image, (**f**) diffraction patterns of the retained austenite.

Figure 4 shows the TEM images of specimens after CHT and after CHT with subsequent SZT at −196 °C. CHT produces a needle-like martensitic microstructure with a relatively large amount of retained austenite. The width of martensitic needles is typically in the range of 100–200 nm (Figure 4a). The retained austenite formations are located at the interfaces of martensitic domains. Their width range is much wider than that of the martensite: some of the austenite formations have a width of a few tens of nanometres, while others are much greater, as illustrated in Figure 4b. The presence of *γ*_R_ is confirmed by diffraction patterns in Figure 4c. The martensite produced by SZT manifests considerable refinement compared to that developed by CHT. The typical width of martensitic domains ranges between 50 and 150 nm (Figure 4d). It is also shown here that the size of the martensitic domains manifests a great level of variability—there are some coarser domains visible in the micrograph, while in some sites, the domains are much smaller. A much higher amount of dislocations are generated within the martensite, as a result of plastic deformation that takes place during the isothermal hold at cryotemperatures. The retained austenite amount is significantly reduced by application of SZT. Additionally, the size of *γ*_R_ formations is much smaller compared to the state after CHT (Figure 4e). The presence of retained austenite at the interfaces of martensitic domains is confirmed by diffraction patterns in Figure 4f.

**Figure 5:**
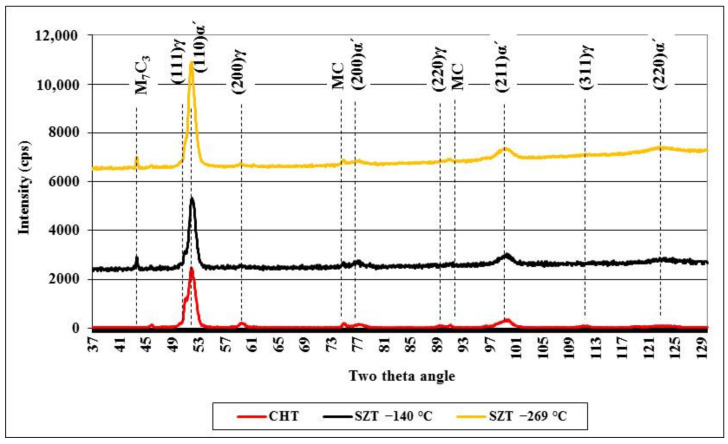
X-ray diffraction spectra of CHT steel and steel after SZT at −140 and −269 °C.

Figure 5 shows X-ray diffraction spectra obtained on the conventionally quenched specimen and on specimens with SZT at −140 and −269 °C. All the spectra contain the peaks of the martensite, retained austenite, and major carbides. In the diffraction profile of the CHT specimen, there are the peaks of (111)γ, (200)γ, (220)γ, and (311)γ, all well-visible. On the other hand, the last two peaks disappear almost completely from the XRD spectra of SZT steel, suggesting a significant reduction of retained austenite amounts after this kind of treatment.

**Figure 6:**
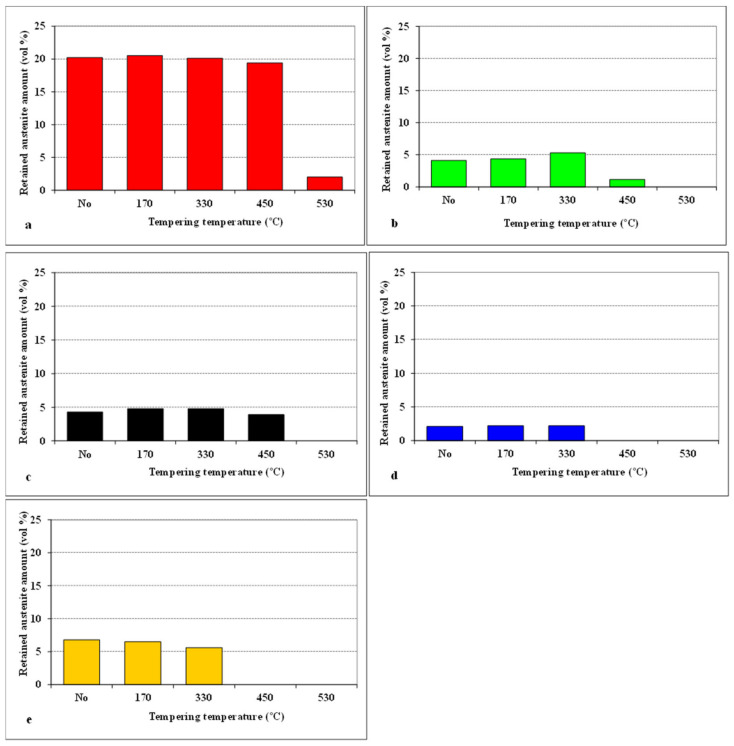
Retained austenite amounts for different SZT specimens, as a function of tempering temperature: (**a**) CHT, (**b**) SZT −75 °C, (**c**) SZT −140 °C, (**d**) SZT −196 °C, (**e**) SZT −269 °C.

Variations in the retained austenite amounts for CHT steel, and for steel samples subjected to different SZTs, as a function of tempering temperature are summarised in Figure 6. It is shown that the application of SZT reduces the *γ*_R_ amount to one tenth—one fourth compared to the state after room-temperature quenching. Tempering at low temperatures does not influence the *γ*_R_ amounts. Alternatively, tempering in the secondary hardening temperature range evokes almost complete retained austenite decomposition in the case of CHT steel. The application of SZT, in addition, accelerates the decomposition of *γ*_R_. Therefore, the amounts of metastable retained austenite lie below the detection limit of XRD after tempering at 450 °C and higher, in the case of the steel SZT in either liquid nitrogen or in liquid helium.

A detailed description of the precipitation behaviour of differently sub-zero-treated Vanadis 6 steel exceeds the scope of the present paper. This topic has been extensively studied recently, and the main results can be summarised as follows: Application of sub-zero treatments evokes acceleration of precipitation of transient M_3_C carbide at low tempering temperatures [19,25]. In CHT steel, the M_7_C_3_ phase precipitates during tempering within the secondary hardening temperature range, while the precipitation of M_7_C_3_ is inhibited in the case of SZT steel, and only growth of M_3_C particles takes place.

### 3.2. Corrosion Behaviour

**Figure 7:**
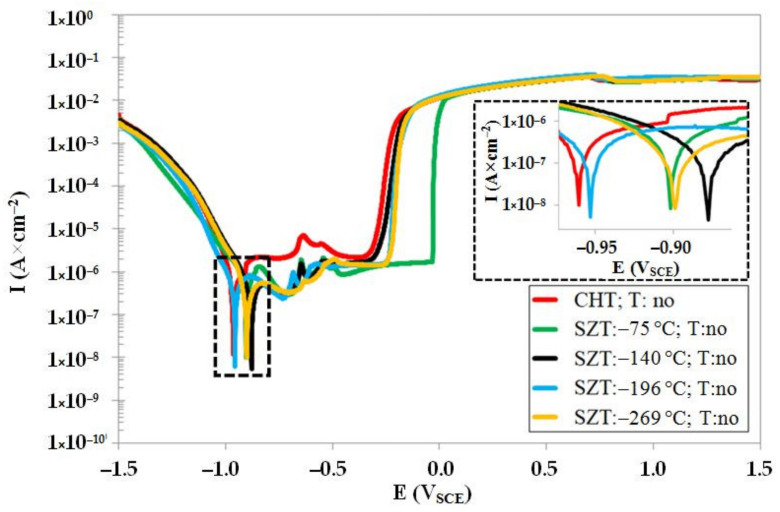
Potentiodynamic polarisation curves of CHT and different SZT specimens in un-tempered state.

Figure 7 summarizes the potentiodynamic curves of CHT specimen and specimens after different SZTs, in un-tempered state. The specimens with SZT at −140 °C manifest the highest corrosion potential (*E_corr_*) (the most anodic), and the *E_corr_* decreases in the order: steel SZT at −269 °C, SZT at −75 °C, SZT at −196 °C, and CHT steel. This suggests that the SZT lowers the steel tendency towards oxidation, and the most convenient SZT temperature for lowering the oxidation tendency was found to be −140 °C. Additionally, the pitting potentials, *E_pit_*, are shifted to the more anodic values (see also Table 3), indicating slightly better resistance of SZT steel to the stable pitting corrosion. As follows from the values of corrosion current, *I_corr_* (Table 3), the CHT specimens manifest the highest corrosion rate, and the corrosion rate (dissolution) decreases in the order: SZT at −75 °C, SZT at −269 °C, SZT at −196 °C, and SZT at −140 °C. These results indicate that the application of SZT improves the corrosion resistance of the Vanadis 6 steel in the prior-to-tempering material state.

**Figure 8:**
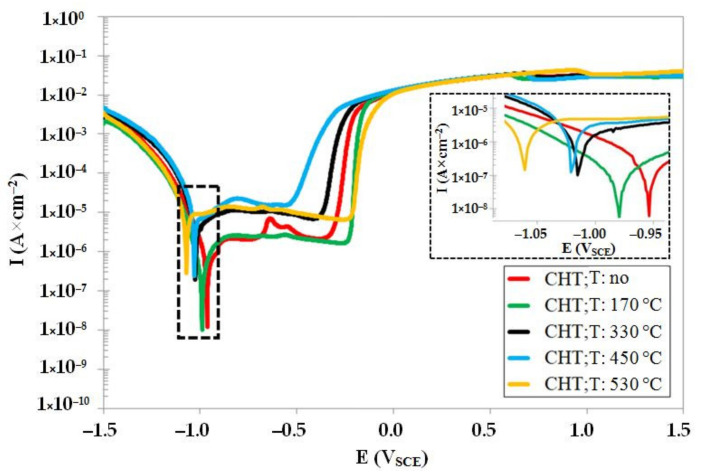
Potentiodynamic polarisation curves of CHT Vanadis 6 steel in un-tempered state and in the states after different tempering treatments.

The potentiodynamic curves of CHT steel in un-tempered state as well as in the states after different tempering treatments are shown in Figure 8. The un-tempered specimen manifests the highest corrosion potential (*E_corr_*), and the *E_corr_* decreases with the increasing tempering temperature. This indicates that the tempering treatment deteriorates the resistance of conventional room-temperature-quenched Vanadis 6 steel against oxidation in 3.5% water solution of NaCl. The variations in the pitting potentials, *E_pit_* (see also Table 4), do not manifest a clear tendency with respect to the level of tempering temperature. The specimens treated at either low (170 °C) or high (530 °C) tempering temperatures manifest more anodic behaviour as compared with un-tempered steel. Conversely, the pitting potentials, *E_pit_*, of specimens tempered at intermediate temperatures indicate higher susceptibility to the stable pitting corrosion. The corrosion current, *I_corr_* (see also Table 4), does not manifest significant changes after tempering at 170 °C. However, it raises rapidly after tempering at 330 °C and higher, suggesting that the corrosion rate of the material increases dramatically. In other words, tempering treatment induces considerable worsening of the corrosion resistance of CHT Vanadis 6 steel.

**Figure 9:**
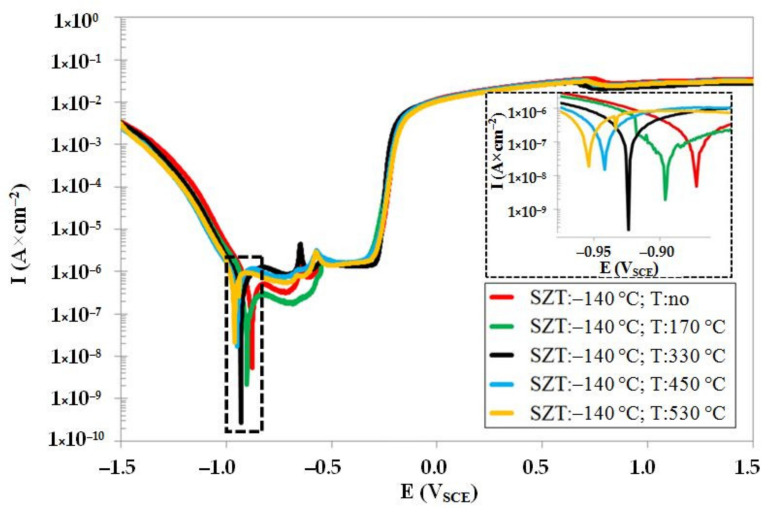
Potentiodynamic polarisation curves of Vanadis 6 steel subjected to SZT at −140 °C, in un-tempered state and in the states after different tempering treatments.

Potentiodynamic curves of the steel that was subjected to sub-zero treatment at −140 °C, un-tempered and tempered at different temperatures, are shown in Figure 9. The *E_corr_* decreases with tempering (in a similar way to CHT steel, see Figure 8). However, it is also obvious that the values of *E_corr_* (for the same tempering regimes) are shifted to higher potentials, suggesting that the SZT improves the resistance of the Vanadis 6 steel towards oxidation, not only in un-tempered state, but also after tempering treatments. The pitting potentials, *E_pit_* (see also Table 5), are practically the same for differently tempered specimens, indicating almost no effect of tempering on the steel susceptibility to the pitting corrosion with SZT at −140 °C. The *I_corr_* follows a very similar tendency to what was recorded for CHT steel. However, it is obvious (see also Table 5) that the values of *I_corr_* are lower than what was recorded for the CHT steel tempered at the same temperatures. It should be noted that similar variations in corrosion characteristics were recorded for other SZTs. One can thus surmise that the SZT makes an overall amelioration of corrosion behaviour of the Vanadis 6 steel when tested in the 3.5 mass % NaCl water solution.

**Figure 10:**
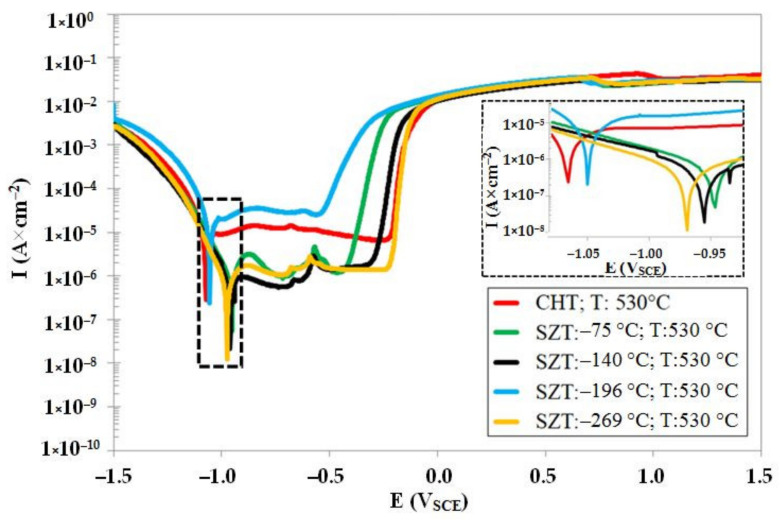
Potentiodynamic polarisation curves of CHT Vanadis 6 steel, and Vanadis 6 subjected to different SZTs, after tempering at 530 °C.

Figure 10 shows the potentiodynamic polarisation curves for the CHT steel specimen and steel specimens after SZT at −75, −140, −196, and −269 °C, after tempering at 530 °C. As shown, the corrosion potential of SZT specimens is higher (more anodic) as compared to the material state after CHT. Further, it is evident that the SZTs at −75, −140, and −269 °C produced higher *E_corr_* than the treatment at −196 °C. The pitting potentials, *E_pit_* (see also Table 6), on the other hand, are shifted to the more cathodic values. This suggests that the application of SZTs provides the examined steel with higher susceptibility to the stable formation of pitting, in the state after tempering at 530 °C. The variations of corrosion current well-follow the changes in *E_corr_*: the samples after CHT have the highest dissolution rate (the highest *I_corr_*), and the *I_corr_* decreases in the order of SZT −196 °C, SZT −75 °C, SZT −140 °C, and SZT −269 °C. Here, a very important finding is that the tendency of the corrosion behaviour improvement, due to the SZT, is partly maintained after tempering at temperatures normally used for the secondary hardening.

**Figure 11:**
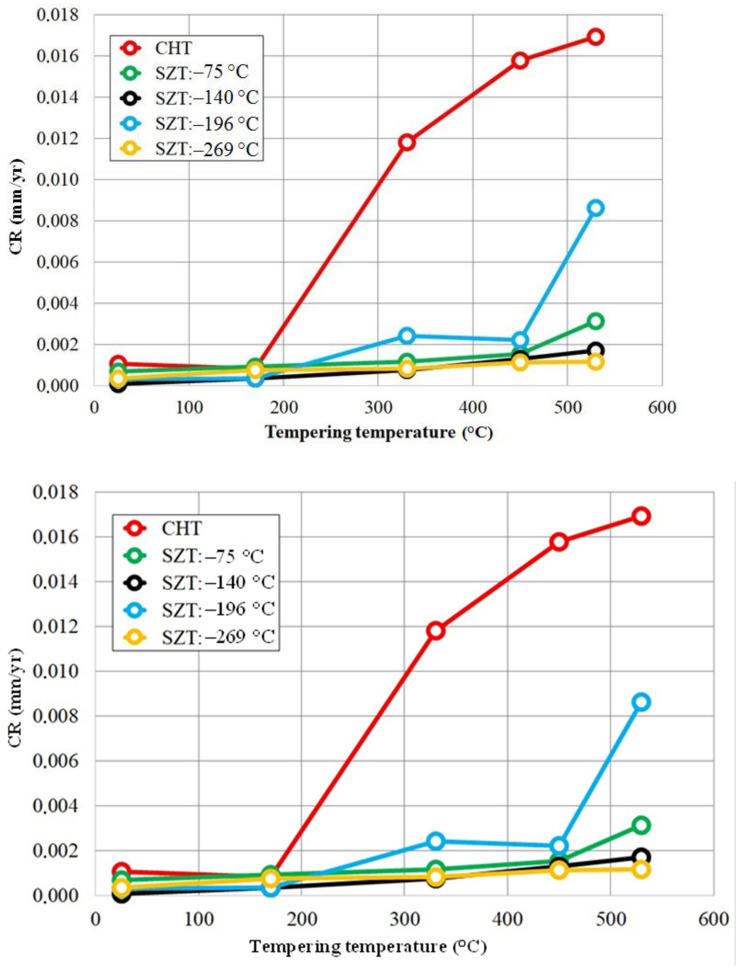
Corrosion rate in dependence on tempering temperature for CHT specimens and for specimens after application of different SZTs.

Figure 11 provides an overview of the dependence of the corrosion rate on the tempering temperature for CHT specimens and for the specimens that were subjected to different SZTs. There are two main tendencies apparently shown. The first one is that the tempering treatment accelerates the corrosion rate (CR), and the higher the tempering temperature, the more accelerated the CR. The second general tendency is that SZTs retard the CR, and that the SZTs at −140 and −269 °C act most effectively in this way.

**Figure 12:**
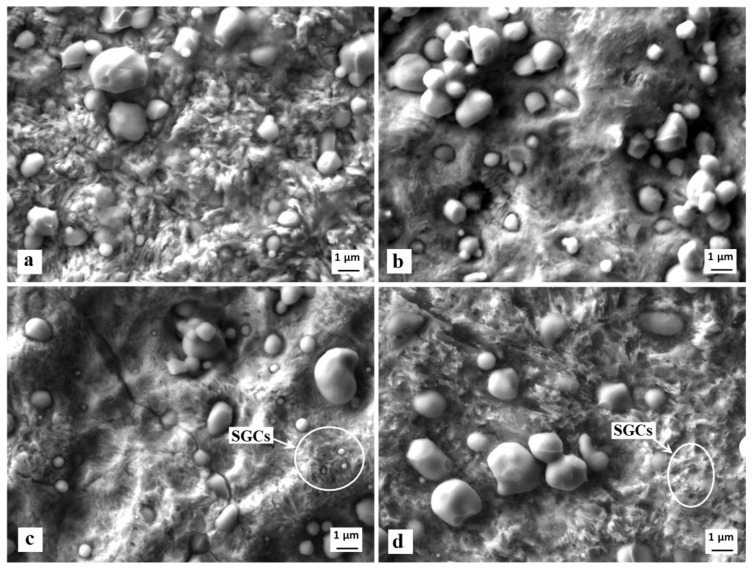
SEM micrographs showing the surfaces after the potentiodynamic polarisation measurements, CHT (**a**), CHT + tempering at 530 °C (**b**), SZT at −140 °C (**c**), SZT at −140 °C + tempering at 530 °C (**d**).

The surfaces after the potentiodynamic measurements of differently heat-treated specimens are presented in Figure 12. It is shown that the carbide/matrix boundaries are preferentially attacked by corrosion. This mainly concerns the boundaries between coarser eutectic and secondary carbides, as they differ from the matrix in terms of their chemistry. The M_7_C_3_ carbides (secondary carbides, SCs), for instance, contain 37.2 ± 0.8 mass % of Cr, 46.4 ± 0.5 mass % of Fe, and 12.7 ± 0.3 mass % of V (there are only metallic elements considered). The eutectic MC carbides are formed mainly by vanadium (73.6 ± 0.9 mass %), but they also contain limited amounts of chromium (9.4 ± 0.5 mass %) and iron (0.8 ± 0.1 mass %). On the other hand, the chromium content in the matrix is around 5.5 mass % only, and that of vanadium is correspondingly much lower (up to 1%). The steel also contains small globular carbides, after SZT in particular, see Figure 3. SEM micrographs in Figure 12c,d clearly delineate that the boundaries between SGCs and matrix are less intensively attacked by corrosion; alternatively, they remain well-embedded in the matrix and assist to lower the corrosion rate at the sites where they are present in sufficiently high amounts.

**Figure 13:**
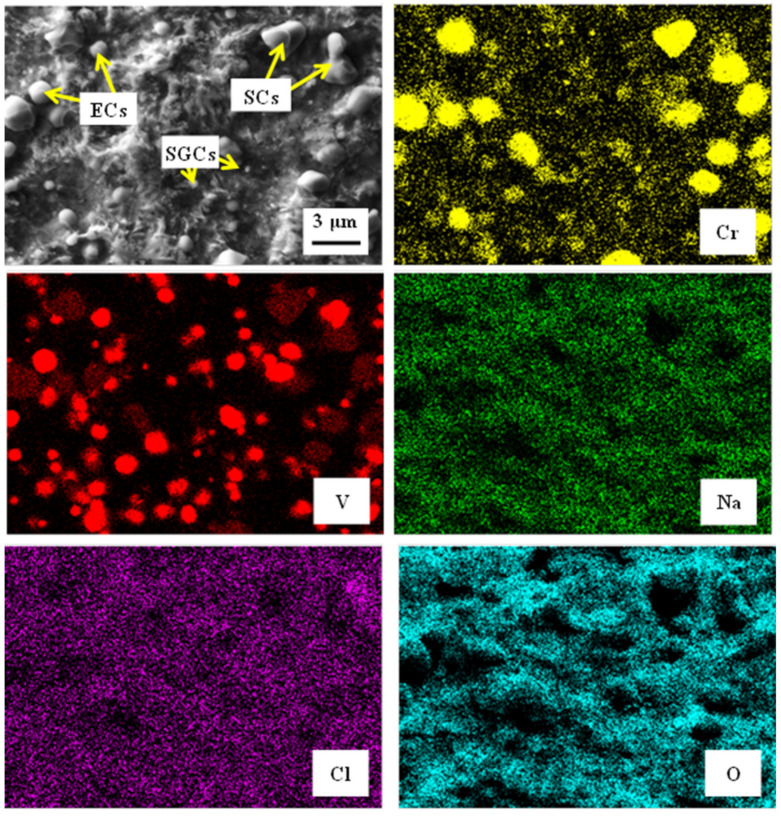
SEM micrograph showing the surface of the specimen that was subjected to the SZT at −140 °C, after the potentiodynamic polarisation measurements, and corresponding EDS maps of Cr, V, Na, Cl, and O.

The SEM image in Figure 13 presents the corrosion-attacked specimen that was subjected to the SZT at −140 °C. The same features as in Figure 12 are visible. This mainly concerns the behaviour of different carbides. These carbides are highlighted in EDS maps of chromium (SCs) and vanadium (ECs). The other EDS maps (chlorine, oxygen, sodium) clearly demonstrate that the corrosion-exposed surface is covered by products of this process. However, it is also seen that the corrosion products’ layer is not uniform. While the matrix is fully covered, the carbides are attacked by the corrosion environment to a much lower extent.

**Figure 14:**
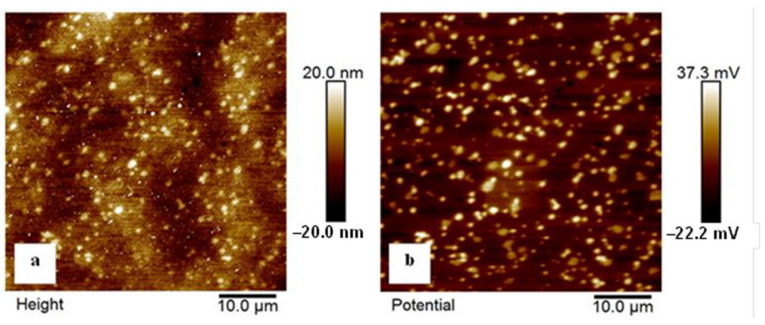
KPFM images of the examined steel sample that was subjected to the SZT at −140 °C. (**a**) Topography and (**b**) work function mapping. Image size 50 × 50 μm^2^.

Figure 14 shows the topography and work function mapping of the phases on the clean surface of the examined steel specimen that was sub-zero-treated at −140 °C. The white spots on the topography image, in Figure 14a, correspond to the carbide particles. The corresponding work function map in Figure 14b undoubtedly confirms that these sites have higher potential than the matrix. The difference is almost fully consistent through the whole measured area, and is about 50–60 mV. In other words, these measurements indicate a more noble behaviour of carbides than the mixture of the martensite and retained austenite (matrix microstructure).

## 4. Discussion

The obtained results infer that the corrosion resistance of the Vanadis 6 steel is generally improved by the application of sub-zero treatments. Additionally, it was demonstrated that ameliorations in corrosion behaviour are maintained after tempering treatment, even though the tempering generally deteriorates the corrosion behaviour. The mentioned variations in corrosion behaviour are the topic of the following discussion.

It has been summarised recently [26] that the application of SZTs evokes a significant reduction of retained austenite amount, refines the martensite, induces an acceleration of the precipitation rate of transient carbides, and produces an enhanced number and population density of small globular carbides.

### 4.1. Retained Austenite Amount and Characteristics

The first factor that makes a clear difference in microstructures of SZTs Vanadis 6 steel and the CHT material is the retained austenite amount. Figure 4 and Figure 6 provide clear evidence on the reduction of this phase in SZT steel, by 70–90%, depending on the temperature of SZT. The mentioned results appear counterintuitive with regard to the corrosion behaviour of the steel at the first glance because it has been experimentally proven that the austenite manifests a more noble behaviour than the ferrite (or martensite) [27,28,29]. The more noble behaviour of the austenite was attributed to a weaker internal stress state and to lower amounts of defects in the gamma phase compared to either ferrite or martensite [30].

However, it was demonstrated recently that sub-zero treatment evokes an introduction of high compressive stresses into the retained austenite of different steels [4,31], due to the combined effect of different thermal expansions of the austenite and the martensite, and to the volumetric effect of the martensitic transformation.

Beneficial effects of compressive residual stresses in the *γ*_R_ on the corrosion resistance can be assumed based upon the obtained results of recent investigations. Peyre et al. [32], for instance, reported increased corrosion performance of the AISI 316L austenitic stainless steel, as a result of compressive stresses that were introduced into the surface by either laser- or shot-peening. Takakuwa and Soyama [33] investigated the effect of various surface finish techniques (and thereby different stress level) on the corrosion resistance of the same steel grade in 5 mass % aqueous solution of H_2_SO_4_, and they arrived at very similar findings. Moreover, they claimed that the principal explanation of improved corrosion behaviour may be based on the fact that the reduction of interatomic spacing due to the compressive stress on the surface facilitates the growth and maintenance of the passivation film. It is also interesting to note that the introduction of compressive stresses enhances the corrosion behaviour not only for ferrous alloys, but, for instance, also for aluminium alloys [34]. Therefore, one can conclude that the high state of compression in the retained austenite contributes to the overall improvement of corrosion resistance of SZT Vanadis 6 steel, even though the *γ*_R_ amount was significantly reduced by this kind of treatment.

### 4.2. Microstructure of the Martensite

Refinement of martensitic domains is the second main consequence of SZT. At the beginning, it should be mentioned that the martensitic transformation that takes place in SZT can be divided into two components: (i) the diffusion-less component that takes place during continuous cooling down from the austenitizing temperature, and (ii) the isothermal component that occurs during the hold at the cryotemperatures. As reported recently [26], the refinement of martensite concerns only the second component, due to spatial limitation effects in the growth of the martensite as well as the result of slow plastic deformation of virgin martensite.

The effect of martensitic domain size on the corrosion behaviour of complex-phase ledeburitic tool steels can only be roughly judged. First, the size of martensite can only be estimated by viewing the TEM micrographs. More exact quantification of this parameter fails, often due to the very small transparent area on thin foils. Additionally, it is clear that the area fraction of refined martensite has a maximum of 17–18%, depending on the extent of the retained austenite reduction due to the particular SZT regime, as illustrated in Figure 7. However, it is logical that the martensitic domains’ boundary density is increased when the domains become smaller, meaning that more domains’ boundaries are present per unit volume. The boundaries of martensitic domains (laths, plates, or needles) will have higher energy levels, as compared to the bulk of the domains, and thereby the finer martensite is expected to be more corrosion-active as compared to the coarse one. Here, it should also be underlined that the refinement of domains concerns only a minor part of the martensite (the isothermally formed one, as mentioned above), and the unfavourable effect of the refinement of martensitic domains on the corrosion performance of SZT steel is hereby significantly reduced.

A clear difference between the martensite produced via CHT and that developed by SZT was seen at the lattice tetragonality level. It is commonly accepted that SZTs reduce the tetragonality of the martensitic lattice of different steels, such as AISI D2 [35] or Vanadis 6 [25]. A logical interpretation is that carbon clusters are formed at dislocations in the martensite during the hold at the cryotemperatures, and that SZT induces an acceleration of precipitation of transient nano-sized cementite particles [14,19]. The carbon atoms in clusters as well as those in precipitates can essentially not contribute to the tetragonality of the martensitic lattice. The carbon content in the martensite of CHT steel was estimated, considering the austenitizing temperature of 1050 °C, carbon contents in major carbides MC and M_7_C_3_ [36], and the level of their dissolution in the austenite at given *T*_A_ [22], to be at around 1.3 wt %. One can expect that the solutionised amount of carbon atoms in the martensite of the steel after SZTs would be correspondingly lower.

Even though a lot of research has been done about the understanding of metallurgical aspects of corrosion behaviour of different steels, only little attention has been paid to the effect of different carbon contents in the martensite, at medium chromium contents, on the corrosion resistance of ferrous materials. There are only two studies devoted to this topic. In the first one, Gulbrandsen et al. [37] reported that the corrosion rate decreased slightly as the carbon content in the martensite rose from 0.095 to 0.12 mass %. In the second study, de Waard et al. [38] established that the addition of up to 2 mass % Cr decreases the effect of carbon content in the martensite on the corrosion resistance to an insignificant level.

However, the Cr content in the matrix of Vanadis 6 steel is at around 5.5 mass % after austenitizing at 1050 °C and quenching, and the results obtained by Gulbrandsen et al. [37] and by de Waard et al. [38] are hardly comparable with the current ones from this point of view. Nevertheless, one can assume a much stronger effect of Cr on the corrosion performance of steels than that caused by carbon (considering the results of de Waard et al. [38], for instance); hence, one can expect almost “no effect” of reduced amounts of carbon atoms solutionised in martensite on the corrosion resistance at 5.5 mass % Cr.

### 4.3. Enhanced Number of Small Globular Carbides

The role of enhanced number and population density of SGCs in the corrosion behaviour of examined steel seems to be a controversial issue. It has been reported in many scientific papers that the presence of carbides, inclusions, or precipitates has a detrimental effect on the corrosion resistance, since there are microelectrochemical cells formed at the carbide/matrix interfaces [39,40]. This was reaffirmed by many authors for ledeburitic steels containing lamellar eutectic mixtures [41], high chromium white-cast irons [42,43,44,45,46], and for Fe-C alloys containing lamellar pearlite [46].

It is obvious from Figure 2 and Figure 3 that SZT increases the amount and population density of small globular carbides. On the other hand, the amounts and population densities of eutectic (ECs) and secondary carbides (SCs) are not affected by SZTs [14]. In Figure 12, it is shown that the areas around the coarser ECs and SCs manifest more distinct corrosion attacks compared to the areas around the SGCs. In addition, it appears that the areas with higher amounts of these small particles undergo corrosion to a lesser extent than the matrix with no presence of SGCs.

Potentiodynamic curves in Figure 7 and Figure 10, and the data in Table 3, provide clear information on the shift of corrosion potential of SZT specimens to higher (more anodic) values, and show that the dissolution rate (corrosion current, *I_corr_*) decreases with the application of SZTs.

For the explanation of “unexpected” ameliorations of corrosion behaviour of SZT steel, it should first be noted that the carbides in experimental materials used in [41,42,43,44,45,46,47] were formed either by the eutectic solidification or by the eutectoid decomposition of the austenite, i.e., at high temperatures where diffusion is possible. Hence, an extensive partitioning of carbon and alloying elements between carbides and solid solutions occurred, which resulted in considerable differences in chemistry between these phases. As a consequence, the galvanic corrosion occurred on the materials’ surfaces due to the difference in corrosion potentials between the carbides and matrix. In the corrosion process, the carbides have a much nobler corrosion potential than the matrix (solid solutions), and hence act as cathodes in galvanic corrosion cells [45]. This is the case of ECs and SCs in the current experimental work. As mentioned above, however, these two carbide types are not influenced by the SZTs, and hence their contributions to the corrosion behaviour can be expected to be invariant to the heat treatment route used. Alternatively, it has been experimentally proven that the SGCs are formed during the hold of the steel at the cryotemperature [1,4,14], where the partitioning of carbon and alloying elements is very limited as there is only little atomic movement at such a low temperature. These particles are a by-product of a more complete martensitic transformation [48]. Additionally, it was indicated that the temperature of −140 °C provides the best balance between the plastic deformation rate of virgin martensite during the isothermal hold of the steel at the cryotemperature and the transformation rate of retained austenite (also takes place at the cryotemperature). Therefore, it is also logical that the presence of SGCs has the most beneficial effect on corrosion behaviour in the case of the steel treated at −140 °C.

**Figure 15:**
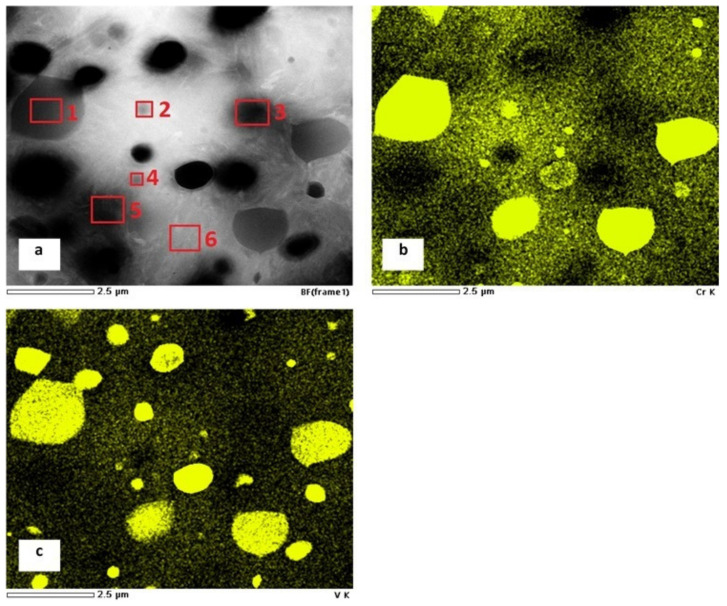
TEM micrograph showing the carbides in martensitic matrix of the specimen after quenching followed by SZT at −140 °C (**a**), EDS map of Cr (**b**), EDS map of V (**c**). The sites of semi-quantitative EDS measurements are labelled and numbered in the TEM image (**a**).

The TEM micrograph in Figure 15a shows different carbides, i.e., the ECs, SCs, and SGCs, in martensitic matrix. In corresponding EDS maps of chromium (Figure 15b) and vanadium (Figure 15c), and also in Table 7, it is shown that the ECs (marked by number 2, and other dark particles) contain much more vanadium than the matrix (marked with number 6). Additionally, it is shown that the SCs differ from the matrix by significantly enhanced chromium content (particle with number 1 as well as two carbides on the right side of the image). On the other hand, the particles numbered 3 and 5 do not manifest any significant partitioning of alloying elements, suggesting that they were formed under diffusion-less conditions.

Therefore, enhanced amount and population density of carbides may not inevitably lead to increasing the overall area ratio of anode (carbides) to cathode (matrix). In addition, an opposite effect can occur, where increased carbides/matrix surface area ratio may contribute to the retardation of corrosion since a more stable protective film on the surface of these carbides can be formed. Experimental investigations of the effect of cementite on the corrosion resistance of carbon steel provided a good example of a much nobler response of cementite on corrosion attacks and confirmed improved corrosion behaviour of the material when coated with Fe_3_C [49].

### 4.4. Precipitation of Carbides

The last difference between the material state after CHT and that after SZTs is an accelerated precipitation rate of nano-sized carbides. According to recent studies [28,50], the precipitation of the Cr-rich M_23_C_6_ carbides during tempering is considered to induce Cr-depleted zones around them, and thereby retards the formation of a protective passive film on the steels’ surfaces. On the other hand, the precipitation of M_3_C carbides with similar Cr content as the matrix has a less detrimental effect on the growth of protective films [28].

For the Vanadis 6 steel, it has been demonstrated recently that the SZT accelerates the precipitation rate of transient cementitic carbides at low tempering temperatures, but these treatments suppress the precipitation of stable M_7_C_3_ phase during tempering in the secondary hardening temperature range [19,25].

Figure 8 and Figure 9 show that the corrosion potential, *E_corr_*, decreases with increasing tempering temperature for CHT steel, as well as for the steel which was subjected to the SZT at −140 °C. It should be mentioned here that the potentiodynamic measurements of specimens subjected to other regimes of SZT (−75, −196, and −269 °C) provided similar qualitative results. Additionally, it is shown (Table 4) that the corrosion current, *I_corr_*, increases rapidly with the tempering, which is clearly reflected in the corrosion rate of different SZT specimens (Figure 11). The mentioned changes in corrosion behaviour characteristics can be ascribed to the precipitation of different carbides and the corresponding changes in the matrix. Only cementitic particles were found in the experimental steel after tempering within the low-tempering temperature range [19,25]. The precipitation of M_3_C does not evoke the Cr depletion of the matrix as the M_3_C contain only very low chromium amount. The only factor that increases the corrosion may be the higher number of activated sites by forming large amounts of M_3_C/matrix boundaries. Increased tempering temperature leads to precipitation of M_7_C_3_ particles in the case of CHT specimens, which reduces the number of solutionised Cr atoms in the microstructure and thereby considerably deteriorates the corrosion characteristics. Instead, the precipitation of M_7_C_3_ carbides was not evidenced after SZTs, and the only consequence of the tempering treatment is the increase in the number of M_3_C particles and their coarsening [19]. Hence, the corrosion characteristics of SZT Vanadis 6 steel are less negatively influenced by high-temperature tempering.

**Figure 16:**
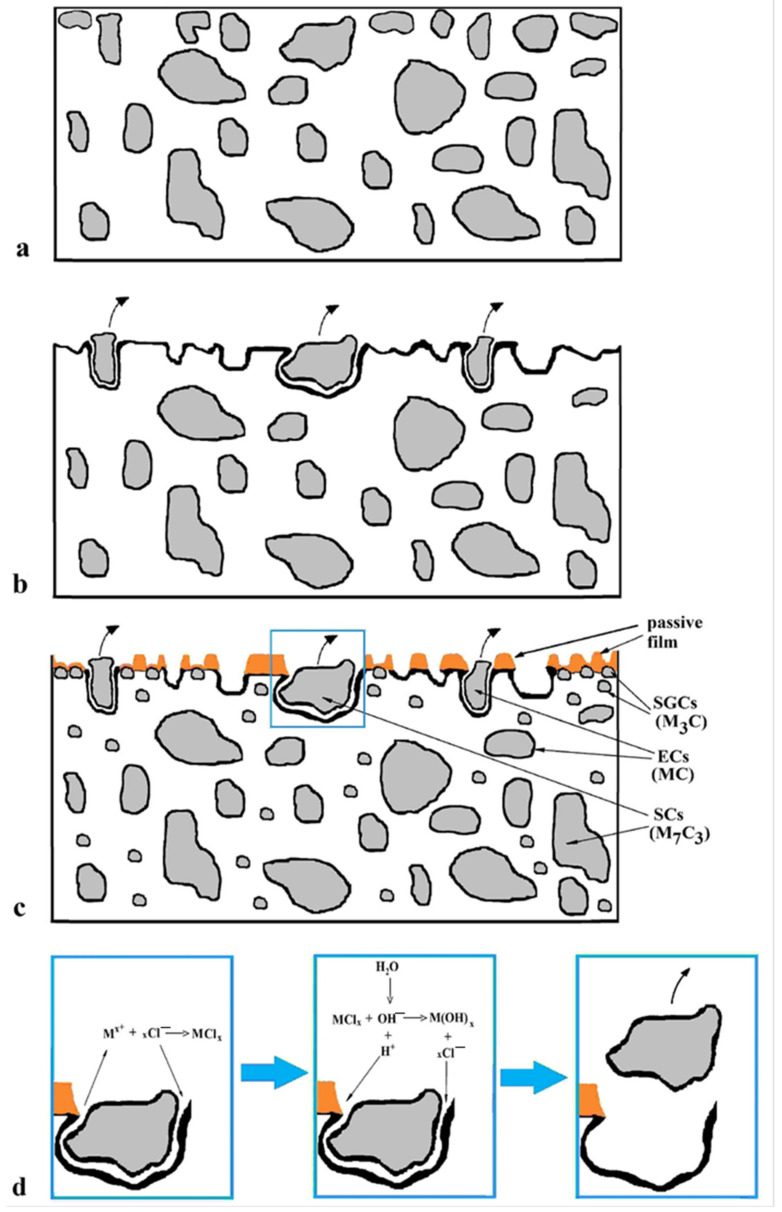
A schematic of the corrosion attack of the Vanadis 6 steel: before testing (**a**), after testing, CHT steel (**b**), SZT steel—overview (**c**), detail from (**d**).

Based on the obtained results, the possible corrosion mechanism of the Vanadis 6 steel in 3.5% NaCl water solution could be delineated. As mentioned above, the steel contains ECs (vanadium-rich, MC), SCs (chromium rich, M_7_C_3_), and certain but very limited amounts of SGCs (Figure 16a). During the corrosion tests, both the ECs/matrix and SCs/matrix interface types are extensively attacked by the corrosion environment, and the carbides are extracted from the surface, which enhances further corrosion (Figure 16b). Additionally, the matrix is considerably attacked by corrosion in this case, as Figure 12a illustrates, and the specimen surface manifests significantly enhanced roughness.

Conversely, the examined steel contains considerably enhanced population density of SGCs after an application of SZTs. The SGCs/matrix interfaces are attacked less extensively by the corrosion environment (Figure 12c and Figure 16c). Moreover, the area percentage of carbides increases at the same time by the application of SZTs. The carbides manifest more noble behaviour than the matrix (Figure 14), and these particles are less covered by the corrosion products (Figure 13). The resulting effect is that the corrosion rate of the SZT specimens is lowered (Figure 11), implying that the application of SZT generally improves the corrosion resistance of the Vanadis 6 steel in 3.5% NaCl water solution.

## 5. Conclusions

The microstructural changes in Cr-V ledeburitic steel Vanadis 6 with different sub-zero treatments and tempering were investigated using SEM, TEM, and X-ray diffraction. The corresponding changes in corrosion resistance in a 3.5% water solution of NaCl were studied by potentiodynamic polarisation tests, calculation of the corrosion rate, and by analysis of attacked surfaces by SEM.

The following conclusions can be expressed from the present study:

1. The austenitizing at 1050 °C for 30 min followed by nitrogen gas quenching produced the microstructure composed of needle-like martensite, retained austenite, and undissolved carbides.
2. Application of sub-zero treatments considerably reduced the retained austenite amount, refined the martensite, enhanced the number and population density of small globular carbides, and modified the precipitation behaviour of nano-sized carbides. The retained austenite amount decreased with the increasing tempering temperature, and it was reduced to values below the detection limit of X-ray diffraction after tempering at 530 °C. Additionally, the number and population density of small globular carbides decreased with tempering in sub-zero-treated steel.
3. In the un-tempered state, the application of sub-zero treatments increased the corrosion potential of the material and inhibited the dissolution. Additionally, sub-zero treatments made the passive films on the surface more stable. These phenomena were most pronounced in the case of the steel treated at −140 °C.
4. Tempering treatment evoked a shift of the corrosion potential to lower values, and at the same time, the corrosion current (dissolution rate) to higher values. For equal tempering temperatures, however, sub-zero-treated steel manifested better corrosion behaviour.
5. The coarse carbide/matrix interfaces were preferentially attacked by corrosion, which resulted in the extraction of these particles, and thereby enhanced corrosion. However, the corrosion attack of the small globular carbides/matrix interfaces was very limited, which significantly contributed to the improvement of the corrosion behaviour of sub-zero-treated material.

## Figures and Tables

**Table 1:** Chemical composition of the experimental steel.

Element (Mass %)	C	Si	Mn	Cr	V	Mo	Fe
**Content**	2.1	1.0	0.4	6.8	5.4	1.5	balance

**Table 2:** The mass percentage, *f*, atomic weight, *W*, most common valance, *n*, electron equivalents of the main elements in the Vanadis 6 steel, *Q*, and calculated electron equivalent of the steel, *Q_total_*, as well as its equivalent weight, *E_w_*.

Element	*f* (Mass %)	*W* (g)	*n* (-)	*Q* = (*n* × *f*)/*W*
C	2.1	12.0107	4	0.699376389
Si	1.0	28.0855	4	0.142422246
Mn	0.4	54.9380	2	0.014561857
Cr	6.8	51.9961	3	0.392337118
Mo	1.5	95.9400	6	0.093808630
V	5.4	50.9415	5	0.530019729
Fe	82.8	55.8450	3	4.448025786
*Q_total_*				6.320551755
*E_W_*				15.821403555

**Table 3:** Corrosion current, *I_corr_*, corrosion potential, *E_corr_*, and pitting potential, *E_pit_*, values acquired from corrosion tests in 3.5 mass % NaCl water solution, for CHT and different SZT specimens in the prior-to-tempering state.

Heat Treatment	*I_corr_* (A·cm^−2^)	*E_corr_* (V)	*E_pit_* (V)
CHT, un-tempered	1.54 × 10^−7^	−0.964	−0.264
SZT −75 °C, un-tempered	9.82 × 10^−8^	−0.903	−0.026
SZT −140 °C, un-tempered	1.02 × 10^−8^	−0.878	−0.221
SZT −196 °C, un-tempered	4.41 × 10^−8^	−0.956	−0.188
SZT −269 °C, un-tempered	4.82 × 10^−8^	−0.901	−0.179

**Table 4:** Corrosion current, *I_corr_*, corrosion potential, *E_corr_*, and pitting potential, *E_pit_*, values acquired from corrosion tests in 3.5 mass % NaCl water solution, for CHT and differently tempered specimens.

Heat Treatment	*I_corr_* (A·cm^−2^)	*E_corr_* (V)	*E_pit_* (V)
CHT, un-tempered	1.54 × 10^−7^	−0.964	−0.264
CHT, tempered at 170 °C	1.21 × 10^−7^	−0.990	−0.189
CHT, tempered at 330 °C	1.74 × 10^−6^	−1.031	−0.283
CHT, tempered at 450 °C	2.32 × 10^−6^	−1.035	−0.395
CHT, tempered at 530 °C	2.49 × 10^−6^	−1.072	−0.142

**Table 5:** Corrosion current, *I_corr_*, corrosion potential, *E_corr_*, and pitting potential, *E_pit_*, values acquired from corrosion tests in 3.5 mass % NaCl water solution, for specimens after SZT at −140 °C and different tempering regimes.

Heat Treatment	*I_corr_* (A·cm^−2^)	*E_corr_* (V)	*E_pit_* (V)
SZT −140 °C, un-tempered	1.02 × 10^−8^	−0.878	−0.221
SZT −140 °C, tempered at 170 °C	5.17 × 10^−8^	−0.902	−0.219
SZT −140 °C, tempered at 330 °C	1.13 × 10^−7^	−0.930	−0.232
SZT −140 °C, tempered at 450 °C	1.88 × 10^−7^	−0.948	−0.230
SZT −140 °C, tempered at 530 °C	2.52 × 10^−7^	−0.961	−0.198

**Table 6:** Corrosion current, *I_corr_*, corrosion potential, *E_corr_*, and pitting potential, *E_pit_*, values acquired from corrosion tests in 3.5 mass % NaCl water solution, for CHT and different SZT specimens, after tempering at 530 °C.

Heat Treatment	*I_corr_* (A·cm^−2^)	*E_corr_* (V)	*E_pit_* (V)
CHT, tempered at 530 °C	2.49 × 10^−6^	−1.072	−0.142
SZT −75 °C, tempered at 530 °C	4.61 × 10^−7^	−0.952	−0.262
SZT −140 °C, tempered at 530 °C	2.52 × 10^−7^	−0.961	−0.198
SZT −196 °C, tempered at 530 °C	1.27 × 10^−6^	−1.065	−0.372
SZT −269 °C, tempered at 530 °C	1.70 × 10^−7^	−0.975	−0.145

**Table 7:** Recorded values of EDS measurements from sites in Figure 16a.

Site No.	Chemical Composition (mass %)				
	Si	V	Cr	Fe	Mo
1	-	12.8	37.8	47.1	2.3
2	-	73.4	17.7	8.9	-
3	0.5	0.5	5.4	92.4	1.2
4	0.2	16.2	7.4	74.8	1.4
5	0.5	0.7	5.7	91.6	1.5
6	0.5	0.6	5.6	92.4	0.9

## Data Availability

The data presented in this study are available on request from the corresponding author. The data are not publicly available due to the fact the this is an ongoing research.
